# Sex differences in patterns of referral and resource utilization in the cardiology clinic: an outpatient analysis

**DOI:** 10.3389/fcvm.2023.1202960

**Published:** 2023-07-31

**Authors:** Lourdes Vicent, Nicolás Rosillo, Guillermo Moreno, Rafael Salguero-Bodes, Clara Goñi, José Luis Bernal, Germán Seara, Héctor Bueno

**Affiliations:** ^1^ Cardiology Department, Hospital Universitario 12 de Octubre, Madrid, Spain; ^2^ Instituto de Investigación Sanitaria Hospital 12 de Octubre (imas12), Madrid, Spain; ^3^ CIBER de enfermedades CardioVasculares (CIBERCV), Madrid, Spain; ^4^ Department of Preventive Medicine, Hospital Universitario 12 de Octubre, Madrid, Spain; ^5^ Facultad de Enfermería, Fisioterapia y Podología, Universidad Complutense de Madrid, Spain; ^6^ Control Management Department, Hospital Universitario 12 de Octubre, Madrid, Spain; ^7^ Centro Nacional de Investigaciones Cardiovasculares (CNIC), Madrid, Spain; ^8^ Facultad de Medicina, Universidad Complutense de Madrid, Madrid, Spain

**Keywords:** cardiovascular disease, gender inequity, cardiology consultations, primary care, symptoms, mortality, sex differences

## Abstract

**Aims:**

Women may have different management patterns than men in specialised care. Our aim was to assess potential sex differences in referral, management and outcomes of patients attending outpatient cardiac consultations.

**Methods and results:**

Retrospective observational analysis of patients ≥18 years referred for the first time from primary care to a tertiary hospital cardiology clinic in 2017–2018, comparing reasons for referral, decisions and post-visit outcomes by sex.

A total of 5,974 patients, 2,452 (41.0%) men aged 59.2 ± 18.6 years and 3,522 (59.0%) women aged 64.5 ± 17.9 years (*P* < 0.001) were referred for a first cardiology consultation. The age-related referral rates were higher in women. The most common reasons for consultation were palpitations in women (*n* = 676; 19.2%) and ECG abnormalities in men (*n* = 570; 23.2%). Delays to cardiology visits and additional tests were similar. During 24 months of follow-up, women had fewer cardiology hospitalisations (204; 5.8% vs. 229; 9.3%; *P* = 0.003) and lower mortality (65; 1.8% vs. 66; 2.7%; *P* = 0.028), but those aged <65 years had more emergency department visits (756; 48.5% vs. 560; 39.9%, *P* < 0.001) than men.

**Conclusion:**

There are substantial sex differences in primary care cardiology referral patterns, including causes, rates, decisions and outcomes, which are only partially explained by age differences. Further research is needed to understand the reasons for these differences.

## GRAPHICAL ABSTRACT

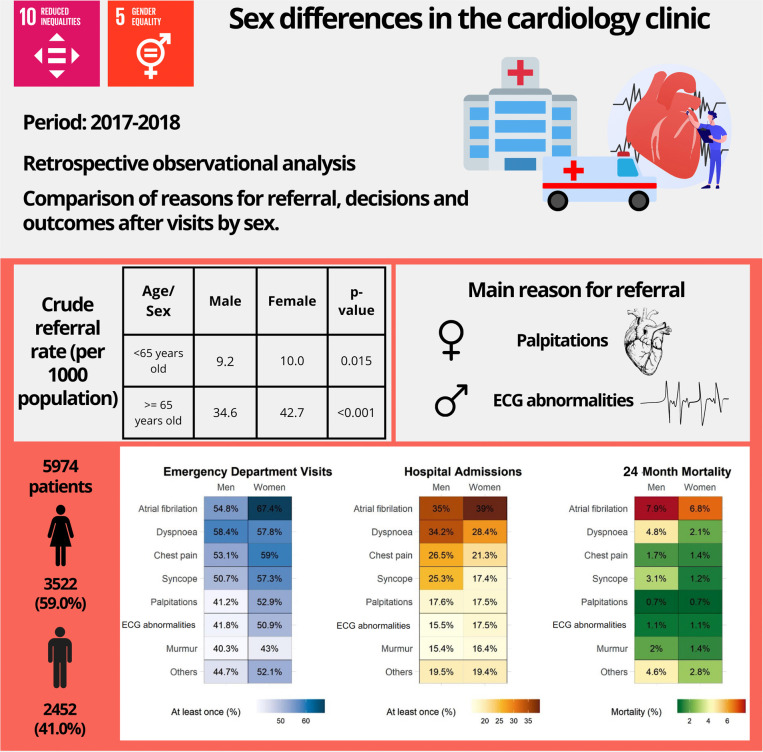
Study design and clinical outcomes. Sex differences in the pattern of referral, complementary examination and outcomes (emergency department visits, hospitalizations, and mortality) in the cardiology consultation.

## Background

Cardiovascular disease (CVD) is the leading cause of death in women (1). However, while cardiovascular mortality has decreased in men in recent years, it has increased in women (2–4). There is a misconception that CVD, particularly coronary heart disease (CHD), is a man's disease. This erroneous assumption has led to inequalities in the care of women with CVD, starting with a lack of initial clinical suspicion and different interpretations of symptoms and signs by women themselves and by those around them (5). Also, different decisions by healthcare professionals for the same cardiovascular signs and symptoms in men and women may lead to inadequate management of CVD in women, both in terms of diagnosis and treatment (6). Higher mortality and poorer outcomes in women have largely been attributed to demographic (older age and life expectancy) and clinical differences, although other factors such as psychosocial stress or adoption of unhealthy habits also play a role (7, 8).

Cardiovascular disease is one of the main reasons for consulting a general practitioner (9); however, the reasons for consulting primary care can vary widely depending on the health care system, geographical location, social class and socioeconomic level (9, 10). To date, most studies evaluating cardiovascular symptoms, such as chest pain or dyspnoea, have been conducted in emergency departments or during hospitalisation (11–13). There are some previous investigations that have looked at the gender differences in the management of specific cardiac symptoms in primary care, particularly chest pain (12, 14). However, there is limited information on the frequency of different symptoms and the reasons for referral from primary care to cardiology.

The aim of this study was to investigate the presence of potential sex differences in the management of the most common cardiological signs and symptoms in outpatient and primary care settings, including differences in referral patterns, management and clinical outcomes.

## Methods

This is a retrospective observational study that included all patients aged ≥18 years referred from primary care for a first cardiology consultation to the outpatient cardiology clinic of the Hospital Universitario 12 de Octubre, a public tertiary hospital belonging to the national health system in Madrid, Spain, using administrative data from primary care referrals and clinical data from the hospital's electronic health record. Exclusion criteria were cases with a previous hospital cardiology history or a previous cardiology consultation, patients who did not attend the medical visit, or cases with missing or inconsistent data. A small number of patients came from outside the hospital catchment area (3.5%). These were included in all analyses as the small numbers should not affect the population rates and calculations.

Consultations were stratified according to the symptom leading to the referral, which was classified by the research team on the basis of the GP's description of the reason for the consultation into 8 main categories: palpitations, dyspnoea, chest pain, ECG abnormalities, syncope, heart murmur, atrial fibrillation and other/miscellaneous.

All patients undergo an ECG at their first consultation. In addition, the consulting cardiologist has an ultrasound machine at his or her disposal. This model of care has been shown to be effective in a previous study (15). All consultations were face-to-face.

The following information was collected: (a) demographic data (sex, age); (b) reason for consultation, as standardised categorical variables; (c) indication for complementary tests or examinations, discharge from consultation or further revision; (d) outcomes during the 24-month follow-up: all-cause mortality, emergency department visits or hospital admissions. Waiting time for cardiology consultation and waiting time for complementary tests were both analysed. A stratified analysis by sex (male and female) and age (over and under 65 years) was performed. The project was approved by the local Research Ethics Committee (CEIm 21/437).

### Statistical analysis

An exploratory descriptive analysis was carried out. Categorical variables were expressed as absolute numbers and percentages, and quantitative ones as mean and standard deviation. Significant differences were assessed using the Chi-square test or the Fisher test in the first case, and the Wilcoxon rank sum test in the second one. Crude attendance rates for cardiology consultations were calculated based on the total reference population of the hospital. All analysis were performed using R software (R Core Team, 2021).

## Results

A total of 5,974 patients (2,452 [41.0%] men; 3,522 [59.0%] women) attended the cardiology consultation as their first cardiology visit between 2017 and 2018. On average, women were older than men (64.5 ± 17.9 vs. 59.2 ± 18.6 years; *P* < 0.001). The catchment area of the hospital is metropolitan and consists of 384,958 individuals aged ≥18 years [202,202 women and 182,756 men (Table 1)], with a medium-low or low socioeconomic status and a high proportion of immigrants (up to 20%, depending on the neighbourhood), mainly born in Latin America, China, Romania and Morocco.

**Table 1:** Crude population referral rates to cardiology consultations by age group and sex (per 1,000 population).

Age Groups	Under 65			Over 65		
Sex	Men	Women	*P*-value^a^	Men	Women	*P*-value^a^
Reference population	152,475	156,293		30,281	45,909	
Referrals by symptom	*n* = 1,403 (9.2%)	*n* = 1,560 (10%)		*n* = 1,049 (34.6%)	*n* = 1,962 (42.7%)	
Chest pain	295 (21%)	286 (18%)	0.065	170 (16%)	292 (15%)	0.3
Population adjusted (per 1,000)	1.9	1.8	0.474	5.6	6.4	0.132
ECG abnormalities	360 (26%)	208 (13%)	<0.001	207 (20%)	248 (13%)	<0.001
Population adjusted (per 1,000)	2.4	1.3	<0.001	6.8	5.4	0.007
Palpitations	222 (16%)	438 (28%)	<0.001	67 (6.4%)	229 (12%)	<0.001
Population adjusted (per 1,000)	1.5	2.8	<0.001	2.2	5.0	<0.001
Dyspnea	101 (7.2%)	137 (8.8%)	0.11	130 (12%)	479 (24%)	<0.001
Population adjusted (per 1,000)	0.7	0.9	0.019	4.3	10.4	<0.001
Syncope	140 (10%)	152 (9.7%)	0.8	152 (14%)	169 (8.6%)	<0.001
Population adjusted (per 1,000)	0.9	1.0	0.720	5.0	3.7	0.003
Atrial fibrillation	31 (2.2%)	20 (1.3%)	0.053	146 (14%)	216 (11%)	0.019
Population adjusted (per 1,000)	0.2	0.1	0.065	4.8	4.7	0.999
Heart murmur	74 (5.3%)	135 (8.7%)	<0.001	75 (7.1%)	158 (8.1%)	0.4
Population adjusted (per 1,000)	0.5	0.9	<0.001	2.5	3.4	0.010
Others	180 (13%)	184 (12%)	0.4	102 (9.7%)	171 (8.7%)	0.4
Population adjusted (per 1,000)	1.2	1.2	0.999	3.4	3.7	0.358
Total	9.2	10.0	0.015	34.6	42.7	<0.001

^a^ Calculated using the Chi-square test.

The mean time from GP referral to cardiology consultation was 48.5 ± 34.1 days in men and 49.5 ± 34.7 days in women (*P* = 0.270). Age-stratified analysis showed that referral to cardiology was higher in women than in men both in patients aged ≥65 years (1,962 women [32.8%] and 1,049 men [17.8%], *P* < 0.001) and in younger patients (1,560 women [26.1%] and 1,403 men [23.5%], *P* = 0.015) [Supplementary Figure S1].

### Reasons for consultation

The most common symptoms presenting to cardiology consultations were palpitations in women (676 patients; 19.2%) and ECG abnormalities in men (570 patients; 23.2%) (Table 1). There were important differences in the reasons for consultation according to age and sex [Table 1, Supplementary Figure S2, S3]. In the younger population (<65 years), palpitations were the most common reason for consultation in women, whereas ECG abnormalities were the most common in men. In the group aged ≥65 years, dyspnoea was the most common situation leading to a cardiology consultation in women, and ECG abnormalities remained the most common in men. Two reasons for referral were most common in women: palpitations (969 patients, 676 women, 69.8%) and dyspnoea (858 patients, 626 women, 72.8%).

### Additional investigations

An electrocardiogram (ECG) was performed in all patients and a bedside echocardiogram was performed in more than half of the patients as part of the initial assessment in the cardiology clinic, with no difference by sex or reason for presentation (1,327 [54.1%] men and 1,944 [55.2%], *P* = 0.573). Additional tests were ordered in 917 (37.4%) men and 1,243 (35.3%) women (*P* = 0.09). The time from the cardiology visit to the performance of the additional tests was 93.5 ± 85.9 days in men and 95.9 ± 103.9 days in women (*P* = 0.474). Supplementary Table S1 shows the most common tests ordered after the consultation by sex and reason for the consultation. Coronary angiography was ordered in 19 [4.1%] men and 8 [1.4%] women with chest pain (*P* = 0.006). Non-invasive testing for ischaemia was performed more often in women aged ≥65 years than in men (239 [34%] vs. 106 [27%], *P* = 0.018). In patients who underwent an ischaemia test, 4 deaths were observed during follow-up, both in the >65 age group (3 in men and 1 in women).

### Follow-up outcomes

Of the total cohort of patients seen in the cardiology clinic, 1,306 men (53.3%) and 1,952 women (55.4%, *P* = 0.062) were discharged to primary care without further tests or visits (men aged ≥65 years were more likely than women to be referred for a subsequent cardiology visit [41.8% men vs. 37.4% women aged ≥65 years (*P* = 0.016)].

**Figure 1:**
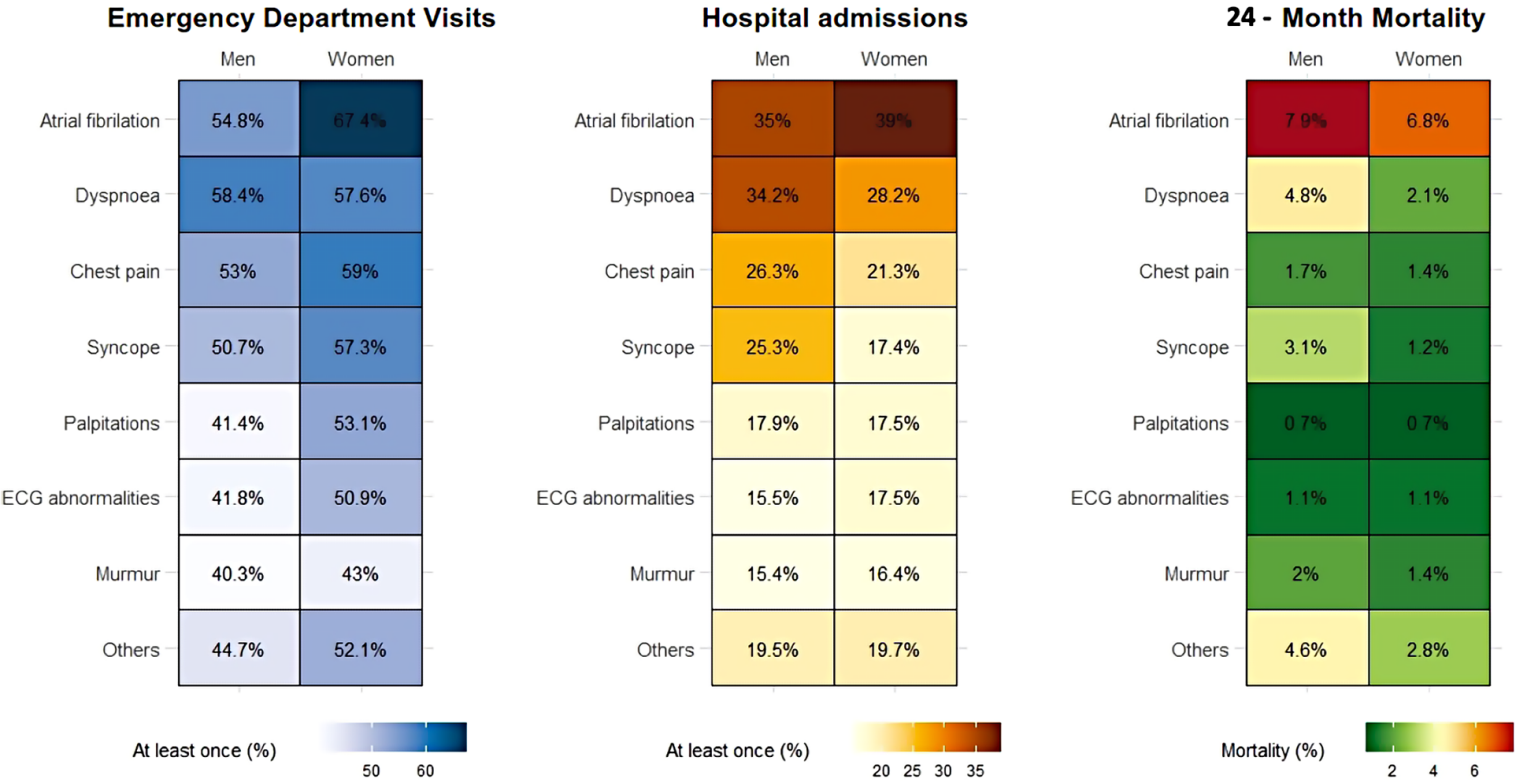
Main clinical outcomes according to sex and reason for consultation from primary care.

During a mean follow-up of 23.7 ± 1.7 months after the cardiology consultation, emergency department visits were more frequent in women (55.0% vs. 47.7%; *P* < 0.001), especially in younger (<65 years) women (48.5% vs. 39.9%; *P* < 0.001; Figure 1, Table 2). In contrast, hospital admissions were more frequent in men aged ≥65 years (22.6% vs. 21.6%, *P* < 0.001), especially to the cardiology department in the overall study population (9.3% vs. 5.8%, *P* < 0.001), but the difference in hospital admissions to the cardiology department between men and women was statistically significant for specific reasons for consultation [chest pain 15.5% vs. 8.8%, *P* < 0.001; and syncope 10.2% vs. 2.5%, *P* < 0.001 (Table 2)]. All-cause mortality was higher in men than in women (2.8% vs. 1.9%, *P* = 0.035). However, this difference was statistically significant only in the group of patients aged ≥65 years (6.0% vs. 3.3%; *P* < 0.001), but not in younger patients (0.2% vs. < 0.1%; *P* = 0.4).

**Table 2:** Outcomes after the first cardiology visit by age, sex and reason for referral.

	<65 years old		*P* value	≥65 years old		*P* value
	Women, *n* (%)	Men, *n* (%)		Women, *n* (%)	Men, *n* (%)	
ECG abnormalities	*N* = 208	*N =* 360		*N* = 248	*N* = 207	
End of cardiology study. Discharge	135 (65%)	246 (68%)	0.4	183 (74%)	134 (65%)	0.036*
Cardiology follow-up	58 (28%)	83 (23%)	0.2	49 (20%)	64 (31%)	0.006*
Death during follow-up	0	1 (0.3%)	>0.9	5 (2%)	5 (2.4%)	>0.9
ED visit	94 (45%)	120 (33%)	0.005*	138 (56%)	117 (57%)	0.9
≥2 ED visits	39 (19%)	61 (17%)	0.6	77 (31%)	66 (32%)	0.8
Hospital admission	20 (9.6%)	38 (11%)	0.7	60 (24%)	50 (24%)	>0.9
Hospitalization in cardiology	4 (1.9%)	17 (4.7%)	0.089	16 (6.5%)	16 (7.7%)	0.6
≥2 hospitalizations	4 (1.9%)	8 (2.2%)	>0.9	18 (7.3%)	19 (9.2%)	0.5
Palpitations	*N* = 438	*N* = 222		*N* = 229	*N* = 67	
End of cardiology study. Discharge	215 (49%)	103 (46%)	0.5	123 (54%)	35 (52%)	0.8
Cardiology follow-up	188 (43%)	100 (45%)	0.6	87 (38%)	27 (40%)	0.7
Death during follow-up	0	0	-	5 (2.2%)	2 (3%)	0.7
ED visit	207 (47%)	84 (38%)	0.021*	146 (64%)	35 (52%)	0.089
≥2 ED visits	118 (27%)	40 (18%)	0.011*	88 (38%)	19 (28%)	0.13
Hospital admission	63 (14%)	25 (11%)	0.3	54 (24%)	26 (39%)	0.014*
Hospitalization in cardiology	20 (4.6%)	13 (5.9%)	0.5	17 (7.4%)	9 (13%)	0.13
≥2 hospitalizations	13 (3%)	9 (4.1%)	0.5	22 (9.6%)	12 (18%)	0.061
Dyspnea	*N* = 137	*N* = 101		*N* = 479	*N* = 130	
End of cardiology study. Discharge	77 (56%)	44 (44%)	0.054	255 (52%)	66 (51%)	0.6
Cardiology follow-up	50 (36%)	50 (50%)	0.044*	189 (39%)	51 (39%)	>0.9
Death during follow-up	0	0	-	13 (2.7%)	11 (8.5%)	0.003*
ED visit	70 (51%)	47 (47%)	0.5	286 (60%)	88 (68%)	0.10
≥2 ED visits	38 (28%)	24 (24%)	0.5	180 (38%)	66 (51%)	0.007*
Hospital admission	14 (10%)	20 (20%)	0.037*	161 (34%)	59 (45%)	0.013*
Hospitalization in cardiology	4 (2.9%)	9 (8.9%)	0.044*	41 (8.6%)	17 (13%)	0.12
≥2 hospitalizations	4 (2.9%)	6 (5.9%)	0.3	45 (9.4%)	28 (22%)	<0.001*
Chest pain	*N* = 286	*N* = 295		*N* = 292	*N* = 170	*P*
End of cardiology study. Discharge	134 (47%)	134 (45%)	0.7	100 (34%)	57 (34%)	0.9
Cardiology follow-up	131 (46%)	137 (46%)	0.9	168 (58%)	94 (55%)	0.6
Death during follow-up	0	0	-	8 (2.7%)	8 (4.7%)	0.3
ED visit	169 (59%)	146 (49%)	0.020*	172 (59%)	101 (59%)	>0.9
≥2 ED visits	97 (34%)	61 (21%)	<0.001*	102 (35%)	60 (35%)	>0.9
Hospital admission	42 (15%)	55 (19%)	0.2	68 (40%)	68 (40%)	0.007*
Hospitalization in cardiology	15 (5.2%)	36 (12%)	0.003*	36 (21%)	36 (21%)	0.028*
≥2 hospitalizations	8 (2.8%)	21 (7.1%)	0.017*	23 (14%)	23 (14%)	0.2
Heart murmur	*N* = 135	*N* = 74		*N* = 158	*N* = 75	
End of cardiology study. Discharge	93 (69%)	41 (55%)	0.052	90 (57%)	32 (43%)	0.041*
Cardiology follow-up	30 (22%)	26 (35%)	0.044*	61 (39%)	41 (55%)	0.021*
Death during follow-up	1 (0.7%)	0	>0.9	3 (1.9%)	3 (4%)	0.4
Consultation in the ED	54 (40%)	27 (36%)	0.6	72 (46%)	33 (44%)	0.8
≥2 consultations in the ED	34 (25%)	15 (20%)	0.4	38 (24%)	17 (23%)	0.8
Hospital admission	17 (13%)	8 (11%)	0.7	31 (20%)	15 (20%)	>0.9
Hospitalization in cardiology	1 (0.7%)	3 (4.1%)	0.13	10 (6.3%)	5 (6.7%)	>0.9
≥2 hospitalizations	3 (2.2%)	5 (6.8%)	0.13	11 (7%)	7 (9.3%)	0.5
Atrial fibrillation	*N* = 20	*N* = 31		*N* = 216	*N* = 146	
End of cardiology study. Discharge	14 (70%)	14 (45%)	0.082	130 (60%)	94 (64%)	0.4
Cardiology follow-up	5 (25%)	14 (45%)	0.15	67 (31%)	47 (32%)	0.8
Death during follow-up	0	1 (3.2)	>0.9	16 (7.4%)	13 (8.9%)	0.6
ED visit	14 (70%)	18 (58%)	0.4	145 (67%)	79 (54%)	0.012*
≥2 ED visits	5 (25%)	10 (32%)	0.6	97 (45%)	47 (32%)	0.015*
Hospital admission	5 (25%)	16 (52%)	0.059	87 (40%)	46 (32%)	0.089
Hospitalization in cardiology	3 (15%)	8 (26%)	0.5	17 (7.9%)	14 (9.6%)	0.6
≥2 hospitalizations	2 (10%)	6 (19%)	0.50	34 (16%)	18 (12%)	0.4
Syncope	*N* = 152	*N* = 140		*N* = 169	*N* = 152	
End of cardiology study. Discharge	96 (63%)	89 (64%)	>0.9	97 (57%)	55 (36%)	<0.001*
Cardiology follow-up	47 (31%)	44 (31%)	>0.9	58 (34%)	81 (53%)	<0.001*
Death during follow-up	0	1 (0.7%)	0.5	4 (2.4%)	8 (5.3%)	0.2
ED visit	76 (50%)	54 (39%)	0.050*	108 (64%)	94 (62%)	0.7
≥2 ED visits	44 (29%)	25 (18%)	0.026*	67 (40%)	57 (38%)	0.7
Hospital admission	17 (11%)	18 (13%)	0.7	39 (23%)	56 (37%)	0.007*
Hospitalization in cardiology	0	5 (3.6%)	0.024*	8 (4.7%)	25 (16%)	<0.001*
≥2 hospitalizations	3 (2%)	3 (2.1)	>0.9	11 (6.5%)	23 (15%)	0.012*
All patients	*N* = 1,560	*N* = 1,403	*P*	*N* = 1,962	*N* = 1,049	*P*
End of cardiology study. Discharge	868 (56%)	769 (55%)	0.7	1,084 (55%)	537 (51%)	0.033*
Cardiology follow-up	577 (37%)	521 (37%)	>0.9	733 (37%)	439 (42%)	0.016*
Death during follow-up	1 (<0.1%)	3 (0.2%)	0.4	64 (3.3%)	63 (6%)	<0.001*
Consultation in the ED	756 (48%)	560 (40%)	<0.001*	1,180 (60%)	609 (58%)	0.3
≥2 consultations in the ED	409 (26%)	265 (19%)	<0.001*	716 (36%)	367 (35%)	0.4
Hospital admission	198 (13%)	199 (14%)	0.2	562 (29%)	356 (34%)	0.003*
Hospitalization in cardiology	49 (3.1%)	97 (6.9%)	<0.001*	155 (7.9%)	132 (13%)	<0.001*
≥2 hospitalizations	42 (2.7%)	63 (4.5%)	0.008*	190 (9.7%)	152 (14%)	<0.001*

*Refers to statistical significance.

ED, emergency department.

Bold values refer to statistical significance.

## Discussion

There are several differences in the management of CVD between women and men. Although previous studies have addressed disparities in acute CV care and preventive therapies, there is little information on the frequency of presentation and management of specific CV symptoms or signs in the outpatient setting according to patients' sex. Our study aimed to assess the presence of gender differences in cardiac care in this setting and suggests that there are differences in cardiac outpatient care, including referral patterns, management of some symptoms and clinical outcomes. In particular, more women than men without a history of CV disease were referred to a cardiologist by general practitioners, most commonly for palpitations in women and for ECG abnormalities in men, with marked differences by age. On the other hand, we did not find any sex differences in the time it took to evaluate the heart and to perform additional tests.

CV causes are one of the main reasons for consulting the general practitioner (9). Previous studies have shown that general practitioners are less aware of CV diseases in women (2, 16, 17), so women are less likely to have access to specialist advice on CVD (18), to have a cardiovascular risk assessment and to be prescribed preventive medication (2). Contrary to previous observations, we found that women from a medium-low income, metropolitan area evaluated by general practicioners have a similar or even higher access to the cardiology specialist in a public tertiary care hospital, even after adjusting for age. However, whether this finding can be extrapolated to other areas is unknown. It has been suggested in a previous study that men seek medical advice later and therefore “under-react” to severe symptoms (19, 20). One of the reasons that could explain the higher frequentation rate among women in the outpatient setting is a greater symptom awareness and disease concern with symptoms of subacute/chronic evolution. It is possible that in the acute context of emergency visits or hospital admissions, women may minimize symptoms due to family pressures. Women are often the carers, they have to do the housework and are responsible for looking after children and relatives, so women may consciously or unconsciously minimize symptoms if they have to return home.

Women consistently use health services more than men, as they have a poorer state of health, poorer quality of life and a greater number of symptoms (21, 22). When considering the differences in the frequency of visits to the primary care physician, it is important to take psychosocial factors into account. A prior study found that a history of affective disorders increased frequentation, as did belonging to socioeconomically disadvantaged areas in the case of women (23). The women in the health care catchment area of our center belong to a medium-low socioeconomic level, and indeed consulted more often with the family physician than men.

### Reasons for consultation

We found significant differences according to patient age and sex. Palpitations are a very common reason for consulting a general practitioner (15% of primary care visits) (24, 25) and the main reason for referral in younger women, whereas it was much less common in men. Palpitations are a benign symptom in the majority of cases, but cause significant discomfort and disability in patients (26, 27). Although palpitations are occasionally related to emotional or psychosomatic causes (>30%) (28), and women with palpitations are even more likely than men to be diagnosed with anxiety disorders (26, 29), in our experience palpitations remain a cause of concern for our primary care physicians and referral for cardiology consultation. Unfortunately, we have not been able to determine the proportion of men and women seen by general practitioners with palpitations who are not referred to cardiology.

Dyspnea was a more frequent cause for referral in older patients, particularly among women. A previous study also reported that women have a higher prevalence and severity of dyspnea than men (30). The reasons for this difference are probably multifactorial, including greater prevalence of obesity, anemia, physical deconditioning, a reduced maximum ventilatory capacity in women and, eventually, a role for emotional factors (30–33). Since dyspnea is an individual and subjective sensation, it is affected by emotional factors (33). Women more commonly suffer from anxiety and mood disorders, and these may worsen the frequency and intensity of dyspnea (33–35). However, dyspnea is an important symptom with prognostic impact since it may reflect underlying heart disease, such as ischemic heart disease, also in women. In a large cohort of patients referred for cardiac noninvasive imaging tests, those presenting with dyspnea had a much higher CV and all-cause mortality than patients without dyspnea (36). As mentioned, dyspnea is more common in women and its assessment should be incorporated into the routine clinical care of these patients, in view of its important prognostic value.

ECG abnormalities were the main reason for consultation in men at all ages. The higher incidence of ECG abnormalities in men compared with women has been described previously (37, 38), and may be partly explained by the higher incidence of cardiovascular disease in men, particularly ischaemic heart disease, which occurs at a younger age (39). The reason for the higher referral rate may be that general practitioners may be more concerned about the presence of structural heart disease in men with ECG abnormalities.

### Additional examinations

Apart from the ECG, which was performed on all patients, and the bedside echocardiogram, which was performed by the attending cardiologist, we found significant differences in the proportion of additional tests ordered, especially in younger patients. Overall, the pattern of additional tests may reflect a greater concern about CVD in men compared with women. One study found that general practitioners considered women to be less likely than men to have ischaemic heart disease, despite having equivalent symptoms or Framingham risk scores to men, and this was associated with a lower indication for diagnostic testing in women (40).

Although the greater use of coronary angiography in male compared with female patients with chest pain and coronary artery disease has been consistently reported for decades (41–45), it is concerning to find this difference 30 years after the description of the Yentl syndrome (41). Gender stereotypes drive differences in the indication for tests such as coronary angiography (43), device implantation (cardioverter defibrillator, cardiac resynchronisation therapy, or mechanical circulatory support) (46), or the performance of invasive procedures such as percutaneous coronary intervention, coronary artery bypass grafting (43), or heart transplantation (47), leading to health inequities. However, it is unlikely that a lower perceived risk of coronary heart disease in women (44) by the consulting cardiologist explains this difference, especially now that the proportion of female cardiologists is high (45% in our department). Rather, this difference may be interpreted as a different perception of patient risk or a higher likelihood of angina with normal coronary arteries in women (45), but an implicit gender bias among cardiologists caring for women with CVD, favouring men who are seen as more robust and willing to take the risk of invasive procedures and interventions than women, has also been described (7, 48). This persistent difference warrants prospective investigation and future corrective action.

Men with ECG abnormalities were more likely than women to undergo a specialised echocardiogram, reinforcing the idea of a higher perceived risk of heart disease in men.

### Clinical outcomes

Although mortality was generally low, there were differences between women and men and in other outcomes. Women, especially younger women and those with palpitations, had more emergency department visits than men. In fact, more than half of the people who go to the emergency department for palpitations are women (49). An alternative explanation for a gender bias in the referral rate and use of additional tests in the palpitations group is that in a tax-funded health care system with universal free access to specialist care at the discretion of the general practitioner, women with palpitations may be more likely to consult a physician. Finally, given that many palpitations are benign and/or non-cardiac in origin, the proportion of true positives among those referred for evaluation may be lower in women than in men. Therefore, the current indication for additional testing could be considered efficient, as fewer tests are indicated in women, who ultimately have fewer hospital admissions and lower mortality. It could also be argued that men are referred to the cardiologist less often than necessary and that this may have a negative impact on hospitalisation and mortality, which are higher in men than in women.

Interestingly, despite having more emergency department visits, women were less likely to be admitted to hospital than men, a finding consistent with previous reports (50). The reasons for this difference are unclear. Given that patients in our cohort did not have previous CV comorbidities requiring specific cardiology consultation, the lower cardiology admission in women is unlikely to be justified by a more favourable clinical profile, so it may be necessary to look at social aspects to explain these differences. A Danish study found similar results, with men having more hospital admissions but higher mortality (19, 20). It has been suggested in previous studies that men seek medical advice later and therefore “under-react” to severe symptoms (19, 20). One of the reasons that could explain the higher frequentation rate among women in the outpatient setting is a greater symptom awareness and disease concern with symptoms of subacute/chronic evolution. It may be possible that women minimized symptoms due to social factors (i.e., family burden, people under their care) particularly in the acute context of emergency visits or hospital admissions. As women most often take most of the housework and are the caregivers for most relatives, they may consciously or unconsciously minimize symptoms or delay in seeking care after they have coped with what they may consider their main responsibilities.

Mortality during follow-up was higher in men. The shorter life expectancy of men is known to be due to a higher incidence of coronary heart disease and cancer (51).

However, CVD in women should not be neglected, as it is also the leading cause of death in women (52), and women also have worse functional status, symptom control and disability than men, known as the “female disadvantage” (6). Despite the fact that CVD is the leading cause of mortality in women, clinicians routinely underestimate the risk of heart disease in them (6, 16, 40, 41, 53, 54). A number of underlying factors lead to inequities in health care between men and women, with a final disadvantage for women (“female disadvantage”). These are found in two main dimensions, The first one, is biological (sex differences), where a lack of understanding of pathophysiological mechanisms and the natural history of CVD in women occurs, with CVD risk assessment being often incomplete and specific female gender factors not taken into account. The second dimension is socio-cultural (gender gap), where stereotypes and social roles of women lead healthcare professionals to consider women less likely to suffer from heart disease, less able to make decisions about their health, and to be treated differently by physicians who routinely treat women (17, 40). Gender biases in the healthcare of CVD start in research, are uncritically transmitted in teaching pathways (health sciences, medical schools), and reproduced in daily clinical practice (17, 40, 53). Interestingly, it has been suggested that women with CVD seen by female cardiologists may have better outcomes than those seen by a male cardiologist (55).

The results of this work help to highlight differences in health care between men and women. Actions are needed at several levels to reduce health inequalities. Healthcare professionals should receive medical training that is sensitive to the differences between men and women. A gender-sensitive medicine approach needs to be promoted to change the medical culture to be more female-friendly, including an increase in the leadership of female cardiologists and other specialists. The socio-economic context of patients needs to be considered in daily clinical practice. More studies are needed to promote women's participation in clinical trials and to deepen the understanding of sex- and gender-related differences and its causes. The empowerment of women as patients should be promoted through education and cultural change that minimise gender stereotypes.

There are some limitations to this study. The reason for consultation was obtained from the GPs' interpretation of the patients' symptoms or reasons for consultation, and the final diagnosis was not available. It was not possible to assess differences in consultation, emergency department visits or hospital admission according to socio-economic variables (education level, income, occupation) because of the lack of such information. Further analysis using this approach is needed to fully understand the complexity of these processes. In addition, our data refer to a specific metropolitan health area, from a tertiary care hospital, so there could be differences in other health areas and other clinics. External validity may be limited and further multicenter analyses are needed. No information was available on the uncertainty of the patients' symptoms.

It is desirable that future studies include sex-specific analyses in order to assess and reduce inequities and the gender gap that is still present in the management of CVD.

## Conclusion

We have identified significant sex differences in patterns of cardiology referral from primary care, including causes, rates, clinical decisions and outcomes, which are only partially explained by differences in age. The reasons for these differences are unclear, and further research is needed to understand the reasons for these differences.

## Data Availability

The raw data supporting the conclusions of this article will be made available by the authors, without undue reservation.
