# Supplementary material for: Sex differences in patterns of referral and resource utilization in the cardiology clinic: an outpatient analysis
Source: Front Cardiovasc Med. 2023 Jul 31;10:1202960. doi: 10.3389/fcvm.2023.1202960 (PMC10425536; doi:10.3389/fcvm.2023.1202960)
Supplement: Supplementary file 1 [file Datasheet1.docx]

**SUPPLEMENTARY MATERIAL**

**Figure S1.** Sex and age distribution of patients referred to the cardiology consultation from primary care


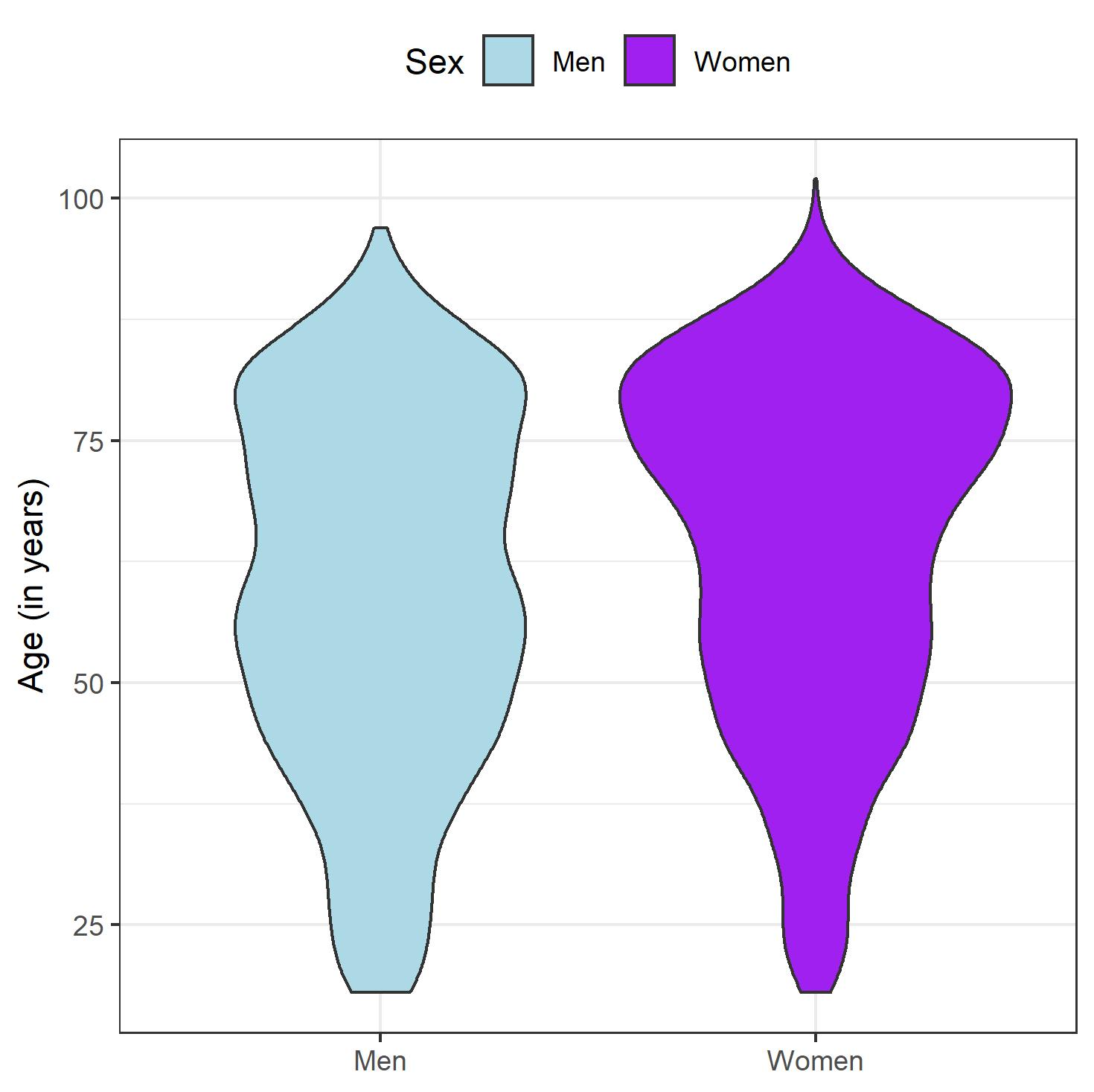


**Figure S2.** Reasons for consultation by sex.

**
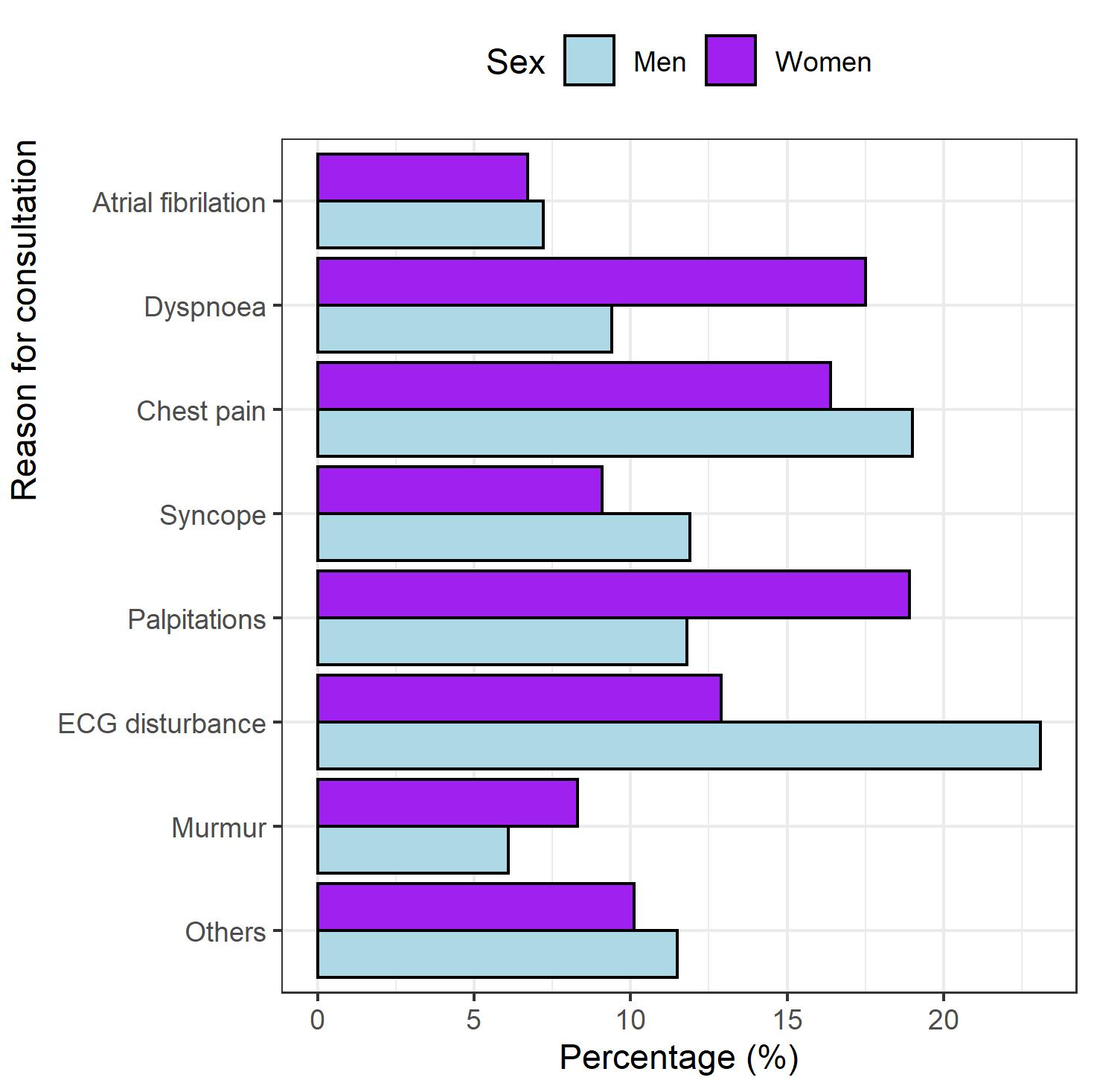
**

**Figure S3.** Main reasons of consultation in cardiology by age and sex.


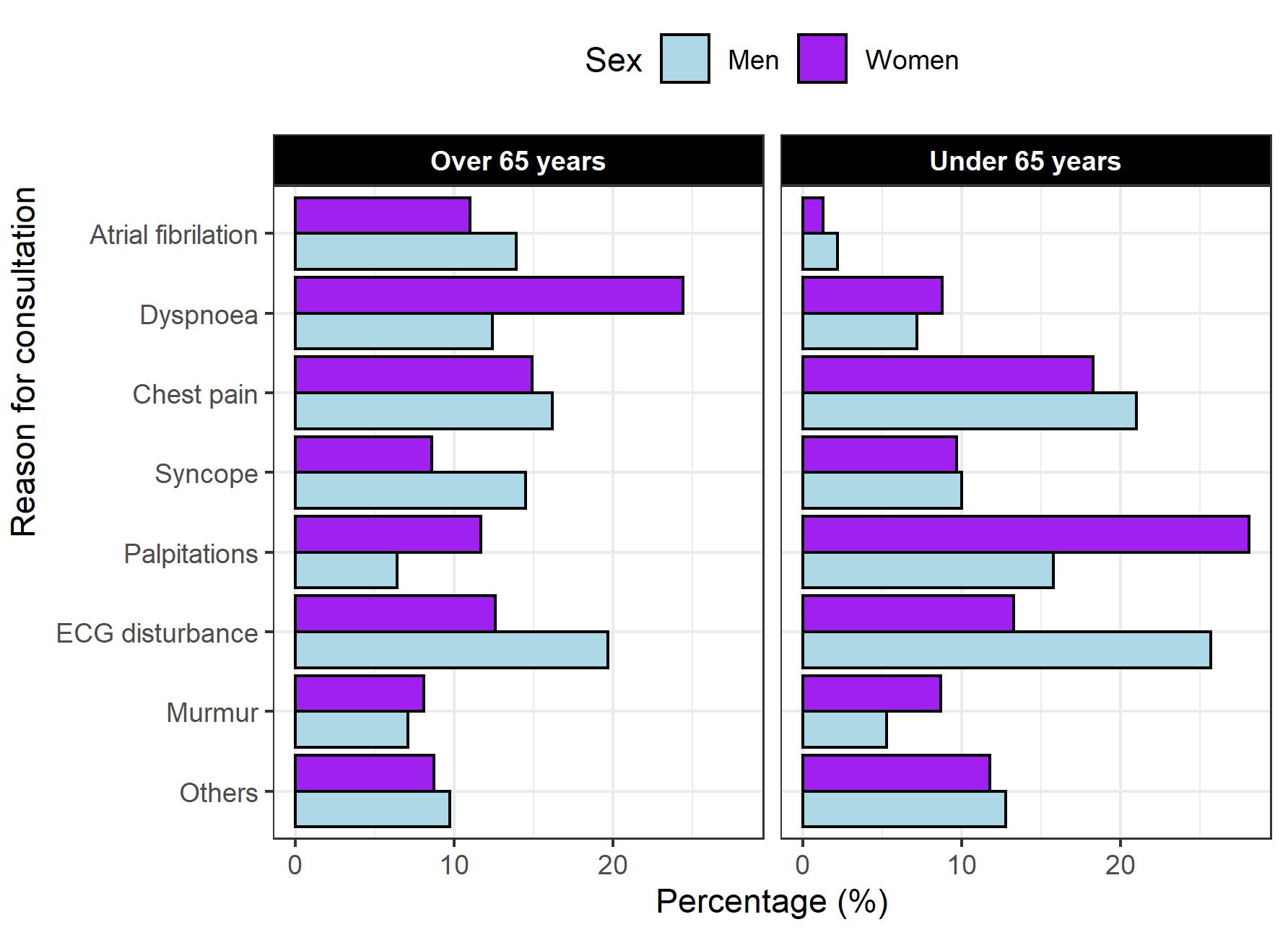


**Table S1.** Most frequently indicated tests by diagnosis after the first cardiology visit by age and sex

|  | **<65 years old** | | **P value** | | **≥65 years old** | | **P value** |
| --- | --- | --- | --- | --- | --- | --- | --- |
|  | **Women n (%)** | **Men n (%)** |  |  | **Women n (%)** | **Men n (%)** |  |
| **ECG abnormalities** | **N= 58** | **N= 88** |  |  | **N=44** | **N=57** |  |
| - Holter - Echocardiography - Non-invasive tests for ischemia - Further imaging tests* - Follow-up visit | 19 (33%)  13 (22%)  19 (33%)  9 (16%)  36 (27%) | 16 (18%)  34 (39%)  20 (23%)  15 (17%)  30 (22%) | | **0.044***  **0.040***  0.20  0.80  0.3 | 16 (36%)  16 (36%)  10 (23%)  4 (9.1%)  49 (29%) | 22 (39%)  22 (39%)  12 (21%)  6 (11%)  22 (24%) | 0.8  0.8  0.8  >0.9  0.4 |
| **Palpitations** | **N=178** | **N=100** | |  | **N=85** | **M=125** |  |
| - Holter - Echocardiography - Non-invasive testing for ischemia - Follow-up visit | 117 (66%)  21 (12%)  39 (22%)  56 (31%) | 52 (52%)  13 (13%)  32 (32%)  33 (33%) | | **0.024***  0.8  0.064  0.8 | 43 (51%)  18 (21%)  19 (22%)  31 (36%) | 10 (40%)  8 (32%)  5 (20%)  9 (36%) | 0.4  0.3  0.8  >0.9 |
| **Dyspnea** | **N=50** | **N=54** | |  | **N=183** | **N=44** |  |
| - Echocardiography - Holter - Non-invasive tests for ischemia - Further imaging tests* - Follow-up visit | 7 (14%)  13 (26%)  28 (56%)  6 (12%)  16 (32%) | 14 (26%)  8 (15%)  29 (54%)  4 (7.4%)  21 (39%) | | 0.13  0.2  0.8  0.5  0.5 | 74 (40%)  17 (9.3%)  72 (39%)  18 (9.8%)  67 (37%) | 19 (43%)  2 (4.5%)  16 (36%)  5 (11%)  12 (27%) | 0.7  0.5  0.7  0.8  0.2 |
| **Chest Pain** | **N=131** | **N=136** | |  | **N=168** | **N=91** |  |
| - Echocardiography - Non-invasive tests for ischemia - Holter - Coronary angiography - Follow-up visit | 13 (9.9%)  96 (73%)  10 (7.6%)  1 (0.8%)  36 (27%) | 16 (12%)  102 (75%)  15 (11%)  9 (6.6%)  30 (22%) | | 0.6  0.7  0.3  **0.019***  0.3 | 21 (12%)  114 (68%)  20 (12%)  7 (4.2%)  49 (29%) | 10 (11%)  58 (64%)  5 (5.5%)  10 (11%)  22 (24%) | 0.7  0.5  0.1  **0.034***  0.4 |
| **Heart murmur** | **N=27** | **N=27** | |  | **N=58** | **N=31** |  |
| - Echocardiography - Follow-up visit | 15 (56%)  12 (44%) | 24 (89%)  10 (37%) | | **0.006***  0.6 | 41 (71%)  24 (41%) | 24 (77%)  17 (55%) | 0.5  0.2 |
| **Atrial fibrillation** | **N=4** | **N=14** | |  | **N=54** | **N=39** |  |
| - Echocardiography - Holter - Follow-up visit | 2 (50%)  1 (25%)  3 (75%) | 4 (29%)  1 (7.1%)  5 (36%) | | 0.6  0.4  0.3 | 23 (43%)  18 (33%)  29 (54%) | 26 (67%)  6 (15%)  17 (44%) | **0.022***  0.051  0.3 |
| **All patients** | **N=549** | **N=531** |  | | **N=694** | **N=386** |  |
| - Echocardiography - Holter - Non-invasive testing for ischemia - Further imaging tests* - Coronary angiography | 110 (20%)  203 (37%)  222 (40%)  46 (8.4%)  3 (0.5%)  170 (31%) | 147 (28%)  127 (24%)  221 (41%)  50 (9.4%)  22 (4.1%)  170 (32%) | **0.003***  **<0.001***  0.7  0.5  **<0.001***  0.7 | | 222 (32%)  165 (24%)  239 (34%)  54 (7.8%)  18 (2.6%)  250 (36%) | 135 (35%)  85 (22%)  106 (27%)  33 (8.5%)  20 (5.2%)  131 (34%) | 0.3  0.5  **0.018***  0.7  **0.027***  0.5 |

*Cardiac magnetic resonance, computed tomography
